# Toward Creating a Subsurface Camera

**DOI:** 10.3390/s19020301

**Published:** 2019-01-14

**Authors:** Wenzhan Song, Fangyu Li, Maria Valero, Liang Zhao

**Affiliations:** 1 Center for Cyber-Physical Systems; University of Georgia, Athens, GA 30602, USA; fangyu.li@uga.edu (F.L.); maria.valero@uga.edu (M.V.); 2 Division of Math and Computer Science, University of South Carolina Upstate, Spartanburg, SC 29303, USA; lzhao2@uscupstate.edu

**Keywords:** subsurface camera, geophysical sensor network, subsurface infrastructure security, distributed computing, real-time in situ imaging

## Abstract

In this article, the framework and architecture of a Subsurface Camera (SAMERA) are envisioned and described for the first time. A SAMERA is a geophysical sensor network that senses and processes geophysical sensor signals and computes a 3D subsurface image in situ in real time. The basic mechanism is geophysical waves propagating/reflected/refracted through subsurface enter a network of geophysical sensors, where a 2D or 3D image is computed and recorded; control software may be connected to this network to allow view of the 2D/3D image and adjustment of settings such as resolution, filter, regularization, and other algorithm parameters. System prototypes based on seismic imaging have been designed. SAMERA technology is envisioned as a game changer to transform many subsurface survey and monitoring applications, including oil/gas exploration and production, subsurface infrastructures and homeland security, wastewater and CO_2_ sequestration, and earthquake and volcano hazard monitoring. System prototypes for seismic imaging have been built. Creating SAMERA requires interdisciplinary collaboration and the transformation of sensor networks, signal processing, distributed computing, and geophysical imaging.

## 1. Introduction

**Figure 1:**
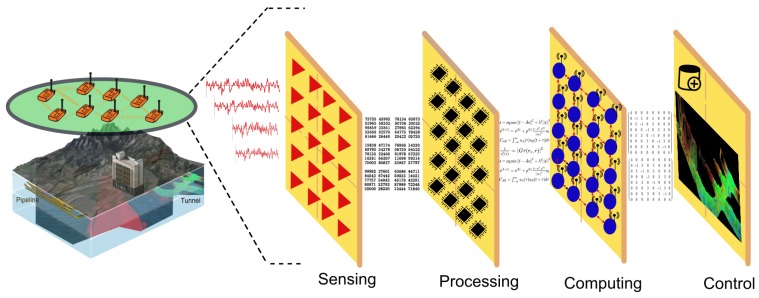
Subsurface Camera (SAMERA) system architecture: sensing, processing, computing and control.

In the eighteenth century, the concept of the optical camera was conceived. The basic mechanism is light rays reflected from a scene enter an enclosed box through a converging lens, and an image is recorded on a light-sensitive medium (film or sensor); a display, often a liquid-crystal display (LCD), permits the user to view the scene to be recorded and adjust settings such as ISO speed, exposure, and shutter speed. In this article, the concept of a Subsurface Camera (SAMERA) is envisioned and described for the first time. The basic mechanism (Figure 1) is as follows: geophysical waves propagating/reflected/refracted through the subsurface enter a network of geophysical sensors where a 2D or 3D image is computed and recorded; control software with a graphical user interface (GUI) can be connected to this network to visualize computed images and adjust settings such as resolution, filter, regularization, and other algorithm parameters.

A SAMERA is a geophysical sensor network that senses and processes geophysical waveform signals and computes a 3D subsurface image in situ in real time. Just as a camera can become a video camera to record a sequence of images, SAMERA can generate a time slice of subsurface images and enable searching, identifying, and tracking underground dynamics for security and control applications. Just as flashlights may be added to an optical camera to enlighten the scene, geophysical transmitters may be added to a SAMERA to illuminate the subsurface for faster image generation and finer resolutions, not merely relying on passive natural events (such as earthquakes). Geophysical transmitters may be add-ons to receivers (e.g., sensors) and convert receivers to transceivers. For example, the seismic exploration of the oil/gas industry often uses explosives or vibroseis to generate active seismic waves, and Ground Penetrating Radars (GPRs) equip active electromagnetic-wave transmitters. Geophysical transmitters can transmit waves at different wavelengths to enable subsurface imaging at different ranges and resolutions. Waves with a longer wavelength typically propagate deeper and further but generate lower-resolution images. If each geophysical transceiver is installed on mobile robots, it would enable a mobile and zoomable SAMERA. Given an area, geophysical transceivers can first spread out to form a sparse array to transmit waves with longer wavelength and generate a coarser subsurface image; if an interested region is identified from the coarser image, the geophysical transceivers can gather closer to form a dense array to transmit waves with shorter wavelength and generate finer subsurface images.

SAMERA technology is envisioned as a game changer to transform many subsurface survey and monitoring applications, including oil/gas exploration and production, subsurface infrastructures and homeland security, wastewater and CO_2_ sequestration, earthquake and volcano hazard monitoring. System prototypes for seismic imaging have been built (Section 3). Creating SAMERA requires interdisciplinary collaboration and transformation of sensor networks, signal processing, distributed computing, and geophysical imaging.

## 2. System Framework and Architecture

A SAMERA system is a general subsurface exploration instrumentation platform and may incorporate one or more types of geophysical sensors and imaging algorithms based on application needs. Various geophysical sensors and methods have been used for subsurface explorations: seismic methods (such as reflection seismology, seismic refraction, and seismic tomography); seismoelectrical methods; geodesy and gravity techniques (such as gravimetry and gravity gradiometry); magnetic techniques (including aeromagnetic surveys and magnetometers); electrical techniques (including electrical resistivity tomography, induced polarization, spontaneous potential and marine control source electromagnetic (mCSEM) or EM seabed logging; electromagnetic methods (such as magnetotellurics, ground-penetrating radar, and transient/time-domain electromagnetics, surface nuclear magnetic resonance (also known as magnetic-resonance sounding)). For environmental engineering applications, the seismic, EM, and electric resistivity methods are often used. All of these geophysical imaging methods can be implemented as a type of SAMERA with the same system framework and architecture, as illustrated in Figure 1.

For the simplicity of presentation and illustration, the following sections describe SAMERA based on seismic imaging, while the framework, architecture, and algorithms similarly apply to other geophysical sensors and imaging approaches. Choosing seismic imaging as the example is also because seismic methods are widely used in subsurface explorations ranging from meters to kilometers in distance and depth.

### 2.1. Sensing Layer and Hardware Platform

**Figure 2:**
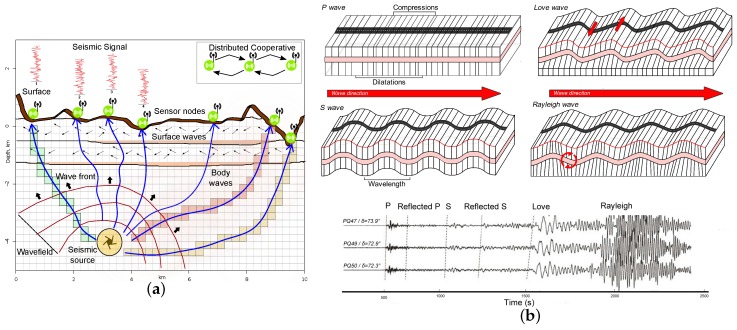
Illustration of seismic imaging. (**a**) Concept. (**b**) Body waves (P and S) and surface waves (Love and Rayleigh).

For seismic imaging, two types of seismic waves are mainly used in the sensing layer: body waves (P and S) and surface waves (Rayleigh wave and Love wave), as illustrated in Figure 2. They have different particle movement patterns [1], resulting in different waveform characteristics and velocities [2]. In Section 3, several seismic imaging algorithms using body and surface waves, respectively, are introduced. Seismometers (often geophones) are used to sense/receive seismic waves. Some seismometers can measure motions with frequencies from 500 to 0.00118 Hz. Deep and large planetary-scale studies often use sub-Hz broadband seismometers, while earthquake and exploration geophysics often use 2–120 Hz geophones. A digitizer is designed to amplify signals, suppress noises, and digitize data via an Analog-to-Digital Converters (ADC) chip. The digitizer of seismic application typically has 16–32 bit resolution, with a sampling rate 50–1000 Hz.

**Figure 3:**
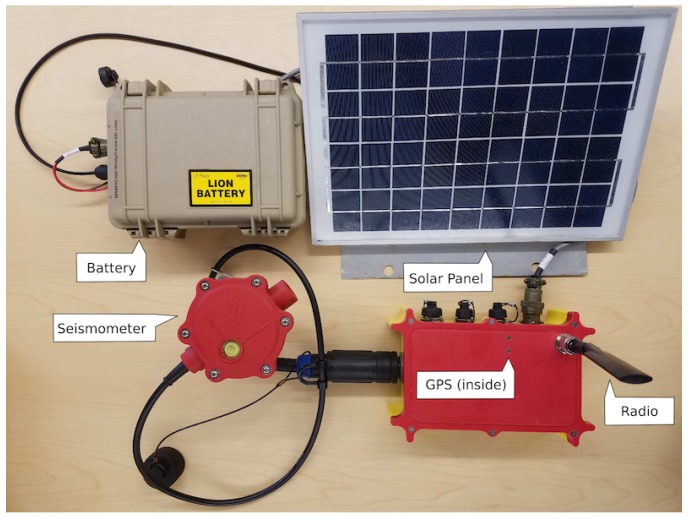
SAMERA hardware platform prototype. It has a geophone, global positioning system (GPS), computing board, wireless radio, solar panel, and battery.

The hardware prototype used for SAMERA (Figure 3) has been designed. The prototype has been used to apply seismic imaging methods. It has a geophone, global positioning system (GPS), computing board, wireless radio, solar panel, and battery. Each sensor nodes equip wireless radio to self-form sensor networks for communication and data exchanges. Sensor networks have been successfully deployed in harsh environments [3,4] for geophysical surveys. GPS provides a precise timestamp and location information for each node. The computing board is Raspberry Pi 3 [5]. It has 1.2 GHz CPU, 1 GB RAM, and GPU for intensive local computing when needed, yet can be put in sleep mode for very low power consumption. Several seismic imaging methods based on this hardware platform have been built and demonstrated as described in Section 3. However, for some geophysical imaging methods (such as Full Waveform Inversion (FWI)), there are concerns on whether they can ever be performed in sensor networks, as they required days’, even months’, computation on IBM mainframes when this article was written. Those concerns appear to be legitimate but will gradually fade out. In the 1970s, an IBM mainframe computer ran at a speed of 12.5 MHz and cost $4.6 million. People in 1970 would similarly doubt a match box-sized board (like Raspberry PI) in 2018 could be 100 times faster than a mainframe and cost only $35. In the past three decades, CPU frequency doubles every 18 months, as predicted by Moore’s law; this trend is expected to continue in the next decade. In addition to expected hardware improvements, Big Data, artificial intelligence, and distributed computing develop quickly as well and become increasingly efficient to deal with those geophysical imaging problems.

### 2.2. Processing Layer

In the processing layer, signal-processing techniques are used to process the raw time-series sensor signals and extract needed information for 2D/3D image reconstruction. The signal-processing functions include data conditioning, noise cancellation, changing point detection, signal disaggregation, cross-correlation, and time and frequency analysis. Most signal-processing tasks are locally performed in each node, while a few processing tasks may need data exchange among nodes, such as cross-correlation used in ambient noise imaging (Section 3.3).

Data conditioning is the very first step of the processing layer, which includes time synchronization [6], data interpolation [7], axis direction initialization [8], and instrumental response removal [9]. Then, to enhance the signals of interest and remove noises, filters, such as the bandpass filter [10], Wiener filter [11], and adaptive filter [12], have been applied. Later, to extract signal event information from the filtered data, wavefield separation [13], body wave (P and S) arrival detection [14], and surface wave source extraction [15] are still drawing more and more attentions.

Recently, Valero et al. [16] applied signal-processing techniques, including taper filter, bandpass filter, local normalization, cross-correlation, stacking, time-frequency analysis, and Eikonal tomography and interpolation to map the shallow seismic surface wave velocity changes. Cali and Ambu [17] also adopted advanced image-processing techniques to obtain more accurate 3D surface reconstruction.

### 2.3. Computing Layer

In the computing layer, the reconstruction of 2D/3D seismic images often involves computations such as linear/nonlinear inversion and optimization, and spatial and temporal stacking. Those computations are traditionally performed in central servers and often need data from all sensors. To implement SAMERA, a key requirement is to perform those computations in sensor networks in real time. Thus, the main research challenge is to develop distributed iterative computing algorithms under network bandwidth constraints. Song et al. [18,19,20,21,22,23,24,25] pioneered the research on in situ seismic imaging in distributed sensor networks. The idea is to let each node compute in an asynchronous fashion and only communicate with neighbors while solving a 2D/3D image reconstruction problem. By eliminating synchronization point and multihop communication used in existing distributed algorithms, the approach can better scale to large numbers of nodes and exhibit better resilience and stability. Randomized gossip/broadcast-based iterative methods have also been used. In these methods, each node asynchronously performs multiple rounds of iterations of its own data, gossips/broadcasts its intermediate result with neighbors for a weighted averaging operation. Iterative computing can be based on first-order and second-order methods (such as distributed ADMM [26] methods). Second-order methods expect to have a faster convergence rate but higher computation cost at each iteration. Each node repeats this process until reaching a consensus across the network.

**Figure 4:**
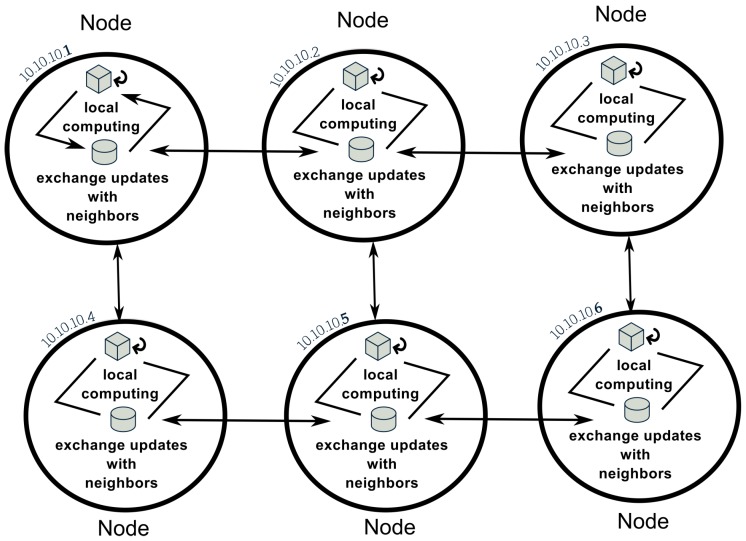
Distributed iterative computing paradigm.

Distributed iterative computing is a paradigm-shifting computing problem and has received much attention [27,28] in the computer science, mathematics, statistics, and machine-learning communities in the past five years. It is advancing rapidly because it is increasingly necessary for many Big Data and Internet of Things applications beyond subsurface imaging. In this new computing paradigm, each node holds a privately known objective function and can only communicate with its immediate neighbors (avoiding multihop if possible). A great effort has been devoted to solving decentralized (fully distributed) consensus optimization problems, especially in applications like distributed machine learning and multiagent optimization. Several algorithms have been proposed for solving general convex and (sub)differentiable functions. By setting the objective function as least-square, the decentralized least-square problem can be seen as a special case of the following problem:

(1) $$\min_{x\in R^{n}}\;F\left(x\right):=\sum_{i=1}^{p}F_{i}\left(x\right)$$

where *p* nodes are in the network and they need to collaboratively estimate model parameters *x*. Each node *i* locally holds the function $F_{i}$ and can only communicate with its immediate neighbors. Figure 4 illustrates this paradigm. The literature can be categorized into two categories: (1) Synchronous algorithms, where nodes need to synchronize the iterations. In other words, each node needs to wait for all its neighbors’ information in order to perform the next computation round. Considering the problem in Equation (1), (sub)gradient-based methods have been proposed [29,30]. However, it has been analyzed that the aforementioned methods can only converge to a neighborhood of an optimal solution in the case of fixed step size [31]. Modified algorithms have been developed in Reference [32], which use diminishing step-size guarantee convergence. Other related algorithms were discussed in [33,34,35,36], which share similar ideas. The D-NC algorithm proposed in [37] was demonstrated to have an an outer-loop convergence rate of $O(1/k^{2})$ in terms of objective value error. The rate is same as the optimal centralized Nesterov’s accelerated gradient method and decentralized algorithms usually have slower convergence rate than the centralized versions. However, the number of consensus iterations within outer-loop is growing significantly along the iteration. (2) Asynchronous algorithms, where nodes do not need to synchronize the iterations. Decentralized optimization methods for asynchronous models have been designed in References [34,38]. The works in Reference [38] leverage the alternating direction method of multipliers (ADMM) for the computation part, and in each iteration, one node needs to randomly wake up one of its neighbors to exchange information. However, the communication schemes in these two works are based on unicast, which is less preferable than broadcast in wireless sensor networks. Tsitsiklis [34] proposed an asynchronous model for distributed optimization, while in its model each node maintains a partial vector of the global variable. It is different from our goal of decentralized consensus such that each node contains an estimate of the global common interest. The first broadcast-based asynchronous distributed consensus method was proposed in Reference [28]. However, the algorithm is designed only for consensus average problem without “real objective function”. Nedic [39] first filled this gap by considering general decentralized convex optimization similar to Equation (1) under the asynchronous broadcast setting. It adopted the asynchronous broadcast model in Reference [28] and developed a (sub)gradient-based update rule for its computation. By replacing (sub)gradient computation with full local optimization, an improved algorithm was designed in terms of the number of communication rounds [40].

For either synchronous or asynchronous algorithms, the design goal is to generate same or near-same results as centralized algorithm with minimal communication cost. The research on distributed iterative computing advances rapidly in the past several years. This section does not intend to survey all methods, but to merely point out some related works and potential direction on computing layer design for the SAMERA.

### 2.4. Control Layer

Control software with a GUI can be connected to this network to view the computed 2D/3D images and adjust system and algorithm parameter settings such as resolution, filter, and regularization parameters. The sensing, processing, and computing layers can execute automatically and autonomously; on the other hand, the control layer allows users to control those layers, such as choose different parameters even different algorithm combinations, to achieve the desired effects. For example, a user may choose to use migration imaging vs. travel-time tomography based on the sensitivity to different types of seismic waves from subsurface properties and the SNR, or the combination with ambient noise imaging to view more or less details at the tradeoff of resource usages. This layer is currently application specific and depends on user preferences, but the user-interface standard will gradually emerge in the future.

## 3. System Prototype Design Examples

This section introduces several SAMERA system prototype design examples based on popular seismic-imaging methods. In each of the following sections, the presentations of processing and computing layers will be emphasized, as they are the main intellectual challenges. The sensing and control layer are more or less engineering and interface issues as described in the previous section.

### 3.1. Travel-Time Seismic Tomography

Travel-time seismic tomography (TomoTT) uses body wave (P and S) arrival times at sensor nodes to derive the subsurface velocity structure; the tomography model is continuously refined and evolving as more seismic events are recorded over time. This method is often used in earthquake seismology, where the event source is a natural or injection-induced (micro)earthquake. TomoTT applies to the scenario where the signal-to-noise ratio (SNR) of body waves is good enough for arrival time picking. Body wave arrival time picking and tomographic inversion are performed in processing and computing layers, respectively.

#### 3.1.1. Processing Layer

When the SNR is relatively acceptable, the arrival-time picking techniques are used to identify the body waves’ (P or S) onset times. Because of the strong background noise in seismic data [41], arrivals are hard to pick or even unidentifiable. The commonly used arrival-picking algorithms are based on statistical anomaly-detection methods including, but not limited to, characteristic function (CF) [14], the short- and long-time average ratio (STA/LTA) [42], Akaike information criterion (AIC) [43], wavelet transform (WT) [44], cross-correlation [45], modified energy ratio (MER) [41], and higher-order statistics (HOS) [46]. In general, the arrival-picking [47] problem can be formulated as the ratio of short-term characteristic function and long-term characteristic function (STCF/LTCF). The characteristic function (CF) [14] can be a kind of statistical metrics, including energy [41], moments [46] and likelihood estimates [48].

Most arrival-picking methods were designed based on single-channel (e.g., vertical axis) seismic data. With triaxis geophones or three-component seismic data, two polarization parameters can be used to distinguish between P and S waves, and noise [49], because wave types and orientations affect the polarization of signal onsets. As the data have three orthogonal ground-motion records corresponding to E, N, and Z, onset polarization could indicate the types (surface or body), phase (P or S) of the wave [11].

#### 3.1.2. Computing Layer

**Figure 5:**
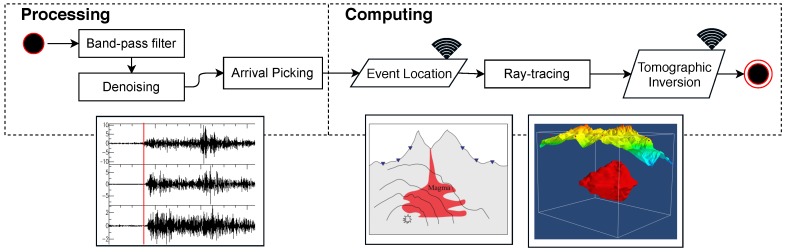
Processing and computing algorithm flows of travel-time seismic tomography. The wireless sign in the figure means communication between nodes is needed.

The picked arrival times are then used to estimate the event source location and origin time in the subsurface, as shown in Figure 5. Thereafter, ray-tracing and tomography inversion are performed. Given the source locations of the seismic events and initial velocity model, ray tracing finds the ray paths from the seismic source locations to the sensor nodes. After ray tracing, the seismic tomography problem is formulated as a large sparse matrix inversion problem. Suppose there are total *M* seismic events and *N* sensors, and *L* cells in the 3D tomography model; then, let $A\in R^{N\dot{M}\times L}$ be the matrix of ray information between *M* events and *N* sensors, $\vec{t}\in R^{N\dot{M}\times 1}$ be the vector of travel time between *M* events and *N* sensors, and $\vec{s}\in R^{L\times 1}$ be the 3D tomography model to calculate. The tomographic inversion problem can be formulated as

(2) $${\vec{s}}^{*}=\arg\min_{\vec{s}}∥\vec{t}-A\vec{s}{∥}_{2}^{2}+\lambda {∥\vec{s}∥}_{2}^{2}$$

where λ be the regularization parameter. In centralized algorithm, the system of equations is solved by sparse matrix methods like LSQR or other conjugate gradient methods [50]. Various parallel algorithms have also been developed to speed up the execution of these methods [51]. However, designed for high-performance computers, these centralized approaches need a significant amount of computational/memory resources and require global information (e.g., $\vec{t}$ and A).

Double-difference tomography [52] was developed to simultaneously solving the event location and three-dimensional tomography model. It claims to produce more accurate event locations and velocity structure near the source region than standard tomography. Its mathematical formulation is in the same format as Equation (2).

#### 3.1.3. Compute TomoTT in Sensor Networks

To implement travel-time seismic tomography in sensor networks, an effective approach is to let each node compute tomography in an asynchronous fashion and only communicate with neighbors. By eliminating synchronization point and multihop communication that are used in existing distributed algorithms, the system can scale better to a large number of nodes, and exhibit better fault tolerance and stability. In the harsh geological field environment, network disruptions are not unusual, and reliable multihop communication is not easy to achieve. Prototype system based on TomoTT [18,19,20,21,22,24] was designed and demonstrated. In Reference [53], a distributed computing algorithm based on vertical partition was proposed. The key idea is to split the least-square problem into vertical partitions, similar to the multisplitting method but being aligned with the geometry of tomography. Later, a node in each partition is chosen as a landlord to gather necessary information from other nodes in the partition and compute a part of tomography. Computation on each landlord is entirely local and the communication cost is bounded. After the partial solution is obtained, it is then combined with other local solutions to generate the entire tomography model. In References [19,25], the block iterative kaczmarz method with a component averaging mechanism [54,55] was proposed. The key idea is that each node runs multiple iterations of randomized kaczmarz, then their results are aggregated through component averaging, and distributed back to each node for next iterations. After multiple iterations, the algorithm converges and generates the tomography. Decentralized synchronous and synchronous methods with random gossip and broadcast [20,40,56] were also developed to solve the inversion problem in Equation (2).

### 3.2. Migration-Based Microseismic Imaging

**Figure 6:**
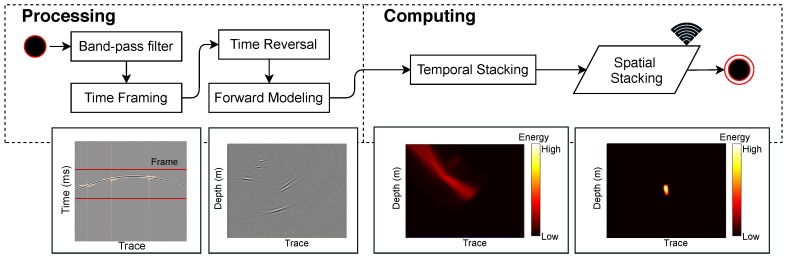
Processing and computing algorithm flows of seismic migration imaging. The wireless sign in the figure means communication between nodes is needed.

Migration-based Microseismic Imaging (MMI) applies reverse-time migration (RTM) principles to locate the microseismic source locations [57,58,59,60]. With a given velocity model, the time-reversed extrapolation of observed wavefields can be calculated based on wave equations. The extrapolated wavefields from different receivers stack together to enlighten the location of seismic sources [61], as illustrated in Figure 6. MMI [61,62,63,64,65] typically uses body waves and has two main steps: forward modeling and stacking (also called imaging condition) in processing and computing layer, respectively.

#### 3.2.1. Processing Layer

In the first step, a bandpass filter with an appropriate bandwidth shall be applied, which is narrow enough to contain signals of interest, and not too narrow to filter out signal components. For active source migration, since sources are controllable and seismic acquisition-array density is high, the raw data and the corresponding wavefields can be completely separated for specific source characterization [58,66]. For passive seismic-source imaging, on the other hand, the determination of a seismic event becomes critical, or else there are not enough time-series data to use for wavefield construction, or computation resources are wasted on background noises [59,60,67]. To extract the window of seismic signal segments containing events, time framing based on energy segmentation can be applied (Figure 6). In addition, for better location, a localized normalization operator is necessary to deal with the issue of unbalanced amplitudes [59].

Let $S(x;t;x_{s})$ denote the source wavefield generated from source location $x_{s}$ and recorded at a spatial location x following the wave equation $\left(\frac{1}{v^{2}\left(x\right)}\frac{\partial^{2}}{\partial t^{2}}-\nabla^{2}\right)S\left(x;t;x_{s}\right)=0$, where *v* is velocity, and $\nabla^{2}$ is the (spatial) Laplacian operator [68]. RTM algorithms use the zero lag of the cross-correlation between the source and receiver wavefields to produce an image $I_{r_{i}}$ at receiver location $x_{r_{i}}$ [58,69]:

(3) $$I_{r_{i}}\left(x,t\right)=S\left(x;t;x_{s}\right)R_{i}\left(x;t;x_{s}\right)$$

Here, $R_{i}\left(x;t;x_{s}\right)$ is the receiver wavefield, which is approximated using a finite-difference solution of the wave equation [68,70,71]. For microseismic imaging, under the virtual source assumption, the source wavefield is eliminated by seismic interferometry using receiver wavefields [60,61,72].

#### 3.2.2. Computing Layer

Equation (3) infers that every receiver generates a 4D wavefield $I_{r_{i}}$. The imaging condition step is to combine all receivers’ wavefields to form the final migration images. It is often produced by summation of wavefields:

(4) $$I\left(x,t\right)=\sum_{i=0}^{N-1}I_{r_{i}}\left(x,t\right)$$

Conventionally, this is done by backward-propagating all data from all sensors at once. Assuming the velocity model is accurate and data contain zero noise, image I(x,t) should have nonzero values only if all the backward-propagated wavefields are nonzero at seismicity location x and time *t*. However, it does not work well with real data with noises. A hybrid imaging condition [59] that was proposed for more effective microseismic imaging is described in Equation (4):

(5) $$I\left(x,t\right)=\prod_{j=0}^{N/(n-1)}\sum_{k=0}^{n-1}I_{r_{j\times n+k}}\left(x,t\right)$$

where *n* is the local summation window length. Length *n* should be selected such that neighboring receivers are backward-propagated together, while far-apart receivers are cross-correlated. Equation (5) requires N/n computations of reverse-time modeling. Notice that the hybrid imaging condition parameter decision needs careful design and evaluation by considering the trade-off between network resource constraints and image quality.

This method is capable of producing high-resolution images of multiple source locations. It adopts the migration imaging principles for locating microseismic hypocenters. It treats the wavefield back-propagated from each individual receiver as an independent wavefield, and defines microseismic hypocenters as the locations where all wavefields coincide with maximum local energies in the final image in both space and time. Microseismic monitoring based on migration imaging is currently considered as the most effective technique to track the geometry of stimulated fracture networks in resource extraction [8].

#### 3.2.3. Compute MMI in Sensor Networks

In sensor networks, temporal stacking and spatial stacking in Figure 6 can be implemented based on Equation (6), which is a slight modification from Equation (5).

(6) $$I\left(x\right)=\prod_{j=0}^{N/(n-1)}I_{c_{j}}\left(x\right)=\prod_{j=0}^{N/(n-1)}\sum_{t}\sum_{k=0}^{n-1}I_{r_{j\times n+k}}\left(x,t\right)$$

In Equation (6), $I_{r_{j\times n+k}}\left(x,t\right)$ is the 4D wavefield from each node, and $I_{c_{j}}\left(x\right)$ is the 3D temporal stacking image of each cluster. In other words, it is a dimension-reduction operation from 4D to 3D. Temporal stacking including summation of wavefields and time axis collapses can be performed in a cluster of sensors [73]. This decreases communication cost in the next step, where spatial stacking is performed between clusters. In spatial stacking, the images of the same location x from different clusters are essentially cross-correlated. The communication cost of passing the 3D image $I_{c_{j}}\left(x\right)$ is still considered expensive for sensor networks. Gaussian beam migration can be further applied to limit the computation and communication to a narrow beam [74], instead of full wavefield $I_{r_{j\times n+k}}\left(x,t\right)$. A primitive prototype of SAMERA on migration-based microseismic imaging has been designed [75]. By using Gaussian beams around these rays, the stacking of amplitudes is only restricted to physically relevant regions. This reduces tens of times of computational and communicational burden without damaging the imaging quality.

### 3.3. Ambient-Noise Seismic Imaging

**Figure 7:**
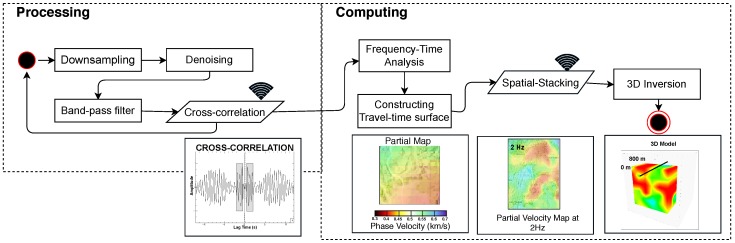
Processing and computing algorithm flows of ambient noise seismic imaging. The wireless sign in the figure means communication between nodes is needed.

To fully utilize the dense seismic array when there are few earthquakes or active sources, Ambient Noise Seismic Imaging (ANSI) [76,77,78] has been developed to image the subsurface using surface waves. ANSI uses radiation from random sources in the earth to first estimate the Greens function between pairs of stations [79,80,81,82] and then invert for a 3D Earth structure [77,78]. Many applications have relied on relatively low-frequency data (in the range of 0.05–0.5 Hz) from ocean noise [83], which images structure in the scale of kilometers, while more local structures can be imaged with higher-order wave modes (higher frequencies) and denser networks [84,85,86]. The main algorithm flows of ANSI (Figure 7) are described as follows:

#### 3.3.1. Processing Layer

This layer is to derive surface-wave travel times through noise cross-correlations. Data conditioning is first applied to raw seismic signals, such as downsampling, denoising, and band-pass filtering. Thereafter, noise cross-correlation $C_{AB}$ between two stations is performed:

(7) $$C_{AB}\left(t\right)=\int_{-\infty }^{\infty }u_{A}\left(\tau \right)u_{B}\left(t+\tau \right)d\tau =\int_{-\infty }^{\infty }\left[-G_{AB}\left(\tau \right)+G_{AB}\left(-\tau \right)\right]d\tau .$$

where $u_{A}$ and $u_{B}$ are the recorded noises at Locations A and B [9]. Theoretical studies have shown that, if the noise wavefield is sufficiently diffusive, the cross-correlation between two stations can be used to approximate the Green’s function $G_{AB}$ between the two sensors or locations [80,87]. By calculating ambient noise cross-correlations between one center station and all other stations, seismic wavefield excited by a virtual source located at the center station can be constructed. Based on noise cross-correlations, the period-dependent surface wave phase and group travel time can be determined between each pair of stations.

#### 3.3.2. Computing Layer

This layer first generates a series of frequency-dependent 2D surface-wave phase-velocity maps, and then 3D inversions are performed across the array to form the final 3D tomography. The eikonal and Helmholtz tomography methods are adopted to determine 2D phase-velocity maps based on empirical wavefield tracking [15,88]. For each event *i*, it directly measures surface-wave phase velocities at each location by the spatial derivatives of the observed wavefield:

(8) $$\frac{1}{c_{i}^{2}\left(r\right)}={\left|\nabla \tau \left(r_{i},r\right)\right|}^{2}-\frac{\nabla^{2}A_{i}\left(r\right)}{A_{i}\left(r\right)\omega^{2}},$$

where τ and *A* represent phase travel time and amplitude measurements, and ${\hat{k}}_{i}≅\nabla \tau \left(r_{i},r\right)c_{i}\left(r\right)$, *c* and ω are direction of wave propagation, phase velocity, and angular frequency, respectively. ${\hat{k}}_{i}$ can be derived directly by solving 2D Helmholtz wave equation, also called eikonal equation, can be derived from Equation (8) under infinite frequency approximation. While the above equations are defined for `events’, it is important to note that the cross-correlation method from Equation (7) effectively turns each station into an ‘event’ recorded at every other station, so the wavefield from virtual sources at each station, as well as the spatial derivatives in Equation (8) can be approximated from the set of cross-correlations with that station. After 2D image reconstruction, the frequency-dependent phase velocities at each location can then be used to invert for vertical profiles. The combination of all 2D models and vertical profiles across the study area produces the final 3D model.

#### 3.3.3. Compute ANSI in Sensor Networks

To compute ANSI in sensor networks, there are two main challenges and can be addressed as follows: (1) The noise cross-correlation step requires every pair of nodes to exchange data with each other at the beginning. Communication cost can be reduced by subsampling, applying a bandpass filter, and limiting the cross-correlation between nodes in near range while approaching far-range cross-correlation through distributed interpolation. (2) The eikonal tomography step requires all nodes to stack their locally calculated velocity maps to form the final 2D/3D subsurface image. The stacking processing can be done through in-network data aggregation or decentralized consensus. Each approach has its own advantages and disadvantages: aggregation works better when the network is reliable, while consensus might be better when network is intermittent. A primitive prototype of SAMERA based on ANSI [16,23,89] has been built and demonstrated.

## 4. Evaluation

**Figure 8:**
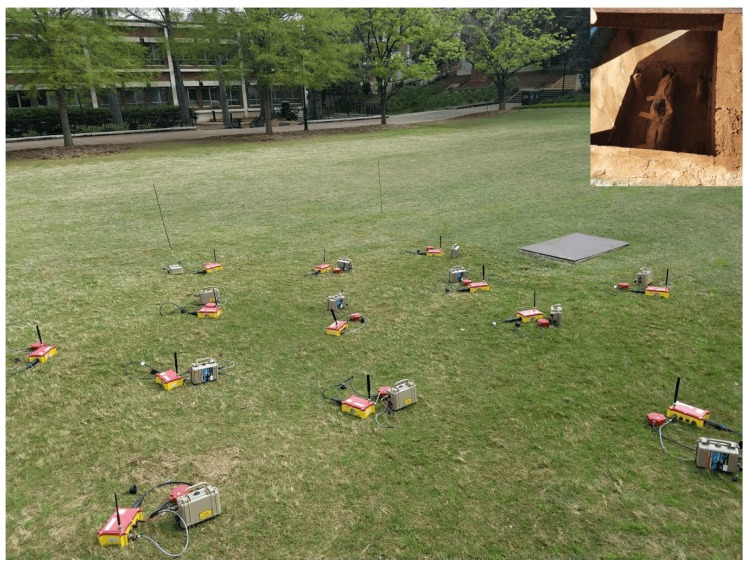
Deployment location at the University of Georgia (UGA) campus. Upper right: A visual of the pipeline that is around ∼1.5 and 1.8 m under surface.

A prototype of the SAMERA system based on ambient-noise imaging principles (Section 3.3) was deployed on the campus of the University of Georgia (UGA). This particular area has an underground pipeline that begins in one building to transport water. Pictures of the area and one of the culvert (where is possible to visualize part of the pipeline) are shown in Figure 8.

The underground pipeline that the system aims to detect is located under the surface at an approximate depth of 1.5–1.8 m. Thirteen seismic nodes were used for this test, and they formed a seismic mesh network for communication and collaboration. The approximate distance between sensor was 3 m; they were located over the area where the pipeline is. The experiment successfully imaged the pipeline under the ground.

### 4.1. Sensing Layer

In the SAMERA design, the sensing layer is responsible of sensing and gathering subsurface waveforms in the seismic network. Our seismic sensors devices were designed and developed to be computation-enabled and energy-efficient.

Every sensor node has a GPS, three channel/component seismometer (geophone), a Raspberry Pi 3 board, a battery, and a solar panel as shown in Figure 3. Some hardware components are housed in a waterproof box called R1+ for protecting them from harsh environments. The low-power GPS interface provides the geolocation of the sensor node and a time stamp is used for the system to collect, synchronize, and process the seismic data. The three-channel geophone is incorporated into the system to detect the velocity of ground movements. Each channel records its own data with respect to its axis N,E, and Z, or directions North, East, and Depth (vertical). The single-board computer (Raspberry Pi) is the core of the system, in charge of collecting and storing data, processing data analytics, communicating with other units and providing raw and processed information to a visualization tool. We also integrated a waterproof battery, 11 V and 99.9 Wh, which can be connected to a 10 Watt solar panel for providing the system with renewable energy.

**Figure 9:**
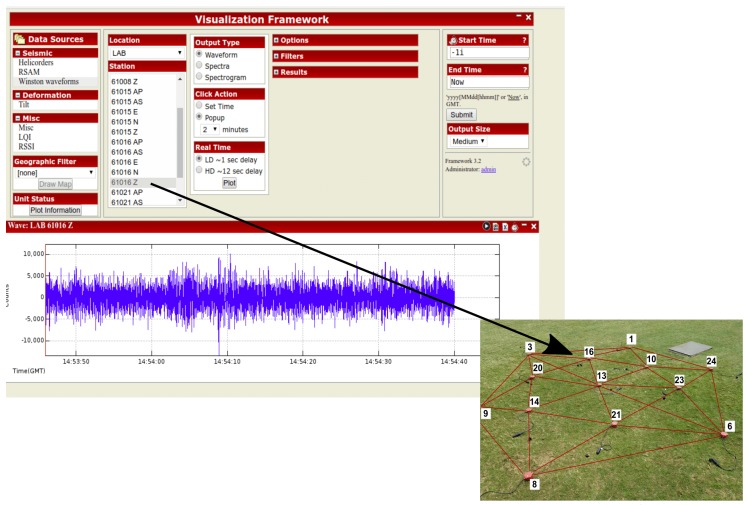
Data visualization. For image purposes, only geophone locations are shown in deployment image.

The detailed specifications of the main single-board computer inside R1 are presented in Table 1. Figure 9 shows an example of data sensed by a seismic node in the field.

### 4.2. Processing Layer

In the processing layer, the system applies signal-processing techniques to process the raw time-series sensor signals and extract needed information for 2D/3D image reconstruction. Once the battery is connected to the sensors, the system automatically calibrates itself and finds a GPS signal for synchronization. System parameters are read from configuration files and the sensor reading from the medium starts. Human intervention is minimal, but the system is manageable via a laptop connected to the mesh network.

**Figure 10:**
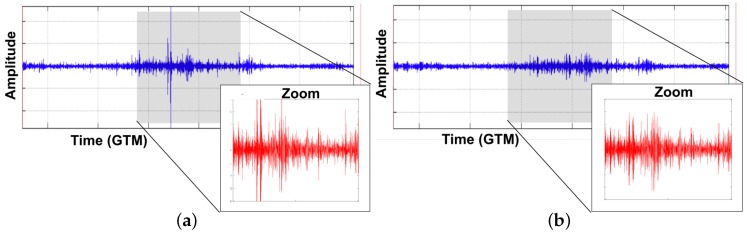
Signal preparation example. (**a**) Raw seismic data sensed by a sensor node. High picks represent possible events that obscure ambient noise. (**b**) Data after preparation preserving ambient noise.

For ambient-noise seismic imaging, the output of this layer consists of a prepared data (after down-sampling and normalization techniques) and the cross-correlation results between sensors in the network. An example of a result of the system data preparation (done by every sensor) is presented in Figure 10.

Figure 10a shows the original raw data that every sensor collects in the sensing layer. Note that the effect of some microseismic events in the gray section. Because ANSI is only based on ambient noise, data preparation in the processing layer is responsible to remove these microevents, normalize the data, and enhance the ambient noise. As it can be seen in Figure 10b, the system effectively removes these unwanted events, and prepares ambient noise for cross-correlation.

**Figure 11:**
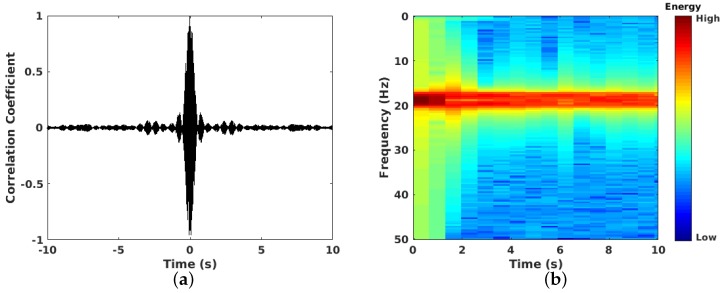
Cross-correlation example between two sensors’ data. (**a**) Casual (positive) and anticasual (negative) symmetric cross-correlation. (**b**) Frequency-time spectrogram where it is possible to distinguish dominant frequencies.

In the processing layer, every node communicates with its neighbor nodes via broadcasting to send the prepared data and perform cross-correlation. Before sending the data, every node applies a band-pass filter to select the spectrum band to be analyzed and a compression technique to improve communication cost between nodes. Once sensor nodes receive the prepared data (and after decompression), they perform the cross-correlation with their own signal. Example of a result of cross-correlation and stacking process (done in distributed fashion) is presented in Figure 11. In Figure 11a, the symmetry cross-correlation result between a pair of stations is presented. As an extra feature, the designed system allows to visualize the cross-correlation result between each pair of seismic sensors. In Figure 11b the frequency-time spectrogram for this cross-correlation is presented. Note the effect of the band-pass filter in the result; this implies that for the final result, only the frequencies between this spectrum band are considered.

**Figure 12:**
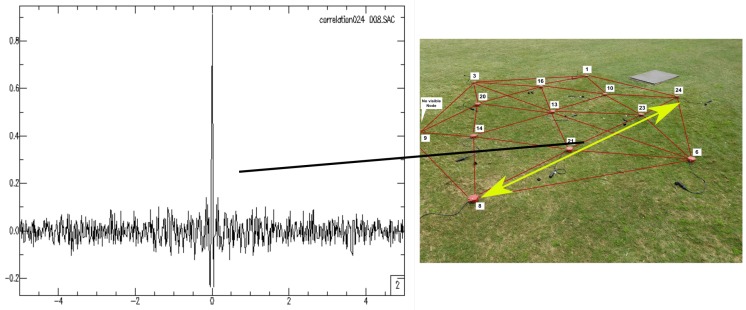
Cross-correlation between a pair of stations, Station 024 and Station 008. For image purposes, only geophone locations are shown in the deployment image.

The system allows the configuration of the frequency band to be analyzed (in a configuration file), which incorporates flexibility to the approach. Figure 12 shows another cross-correlation result after stacking seven hours of cross-correlation

### 4.3. Computing Layer

**Figure 13:**
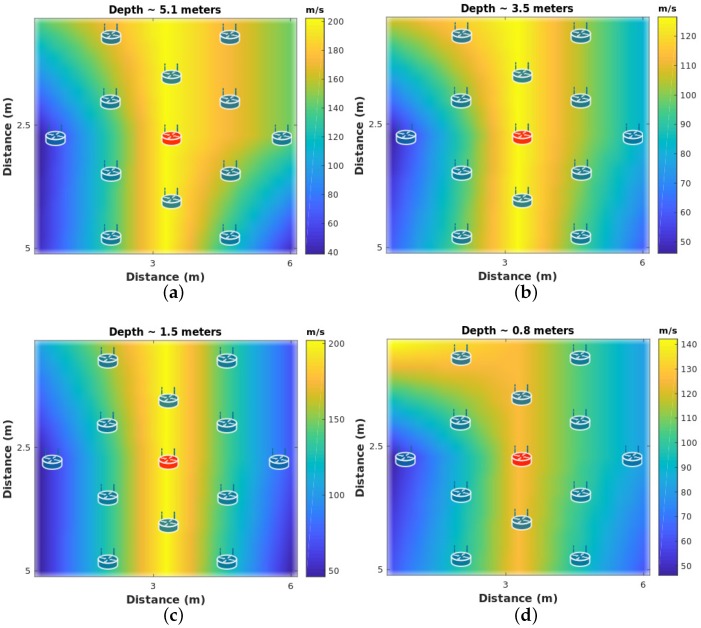
Velocity Maps. (**a**) Layer ∼5.1 m depth. (**b**) Layer ∼3.5 m depth. (**c**) Layer ∼1.5 m depth. (**d**) Layer ∼0.8 m depth. Sensor-node locations are plotted as reference.

For ambient-noise seismic imaging, the output of this layer consists of a series of velocity maps that have been constructed collaboratively by the seismic network. To accomplish this, each node first constructs a velocity estimation based on a frequency-time analysis made to the cross-correlation results. Then, Eikonal tomography is applied to construct a partial travel-time surface. A spatial-stacking process between the seismic sensors is performed to estimate the velocity maps of the area at different depths. An example of velocity maps at different depths is shown in Figure 13.

Figure 13 shows the reconstructed subsurface image by the seismic network, which shows the velocity variation of the subsurface at different depths. A pattern of high velocities at the center of the seismic deployment is evident. This image corresponds to the pipeline that is located under the deployment.

**Figure 14:**
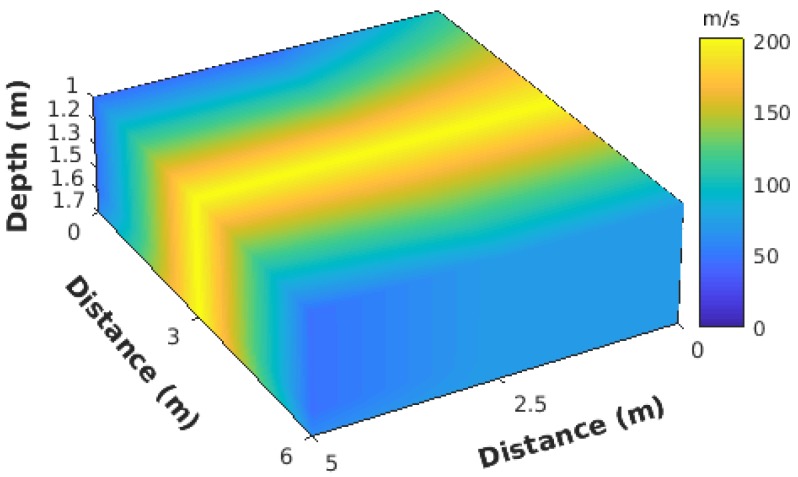
3D velocity subsurface. Layers between 1 and 1.7 m.

The network constructs a 3D subsurface velocity image, as shown in Figure 14, by interpolating the velocity profiles from all the nodes. In this figure, only depths between 1 and 1.7 m are shown. In the center of the velocity map, the high-velocity area corresponds to the pipeline location. Due to the high propagation velocity of the metal pipe, the surrounding soils also show higher velocities than other areas. It seems the horizontal resolution was still not high enough to estimate the real diameter of the pipe, because the frequency range we used in the current application was quite wide. If specific narrow frequency bands, which have the most significant responses with the pipe, are used, the resolution is higher. In addition, vertical resolution can be further improved if there are more stations. This result shows the system is able to see structures under the subsurface and potentially extends the work for some security issues (for example, detecting broken pipelines and detecting tunnels).

### 4.4. Remarks

Pipe detection and location have been demonstrated in this experiment based on the SAMERA framework and architecture. Control software with a GUI was also connected to this network to view the computed 2D/3D images and adjust the system and algorithm parameter settings.

The same system may be used for steam-/water-leakage detection since fluid dramatically reduces seismic propagation velocity. In addition, the leakage location should be along the pipe system, so the high-velocity pipe shape imaging result associated with a low-velocity area can infer the leakage.

## 5. Conclusions

Creating a SAMERA requires interdisciplinary collaboration and the advancement of sensor networks, signal processing, distributed computing, and geophysical imaging. Prototypes based on several seismic-imaging methods have been demonstrated, yet there are many research challenges and opportunities, such as: (1) Full automation: today, geophysical imaging often involves humans in the loop, which is not a surprise as the first generation optical camera is not fully automatic either. For example, the initial velocity model and some algorithm parameters are still based on experience, so the questions are: how to self-learn and self-optimize parameters to make subsurface-imaging computing fully automatic? How to automatically build the initial velocity model? How to integrate machine learning (e.g., data-driven) with physics-based modeling to enable better automation? (2) Fast completion: distributed iterative-computing frameworks and principles have been laid out, yet many practical issues remain to address, such as how to decide the stopping/pausing criteria to avoid overfitting, and how to generate subsurface images faster under the bandwidth constraint and random network failures. (3) Data fusion: different geophysical sensors/methods are sensitive to different subsurface geophysical properties, and the question remains of how to integrate different geophysical methods for joint inversion to generate better subsurface images. These research questions are no longer unique to SAMERA creation and have received much attention in Big Data, machine learning, Internet of Things, and other domains, beyond geophysics, and increasingly better solutions are developed every day. With these recent rapid advancements and cost reduction of both hardware and computing algorithms, it is the right time to start creating the SAMERA—the camera to see through the subsurface.

## Figures and Tables

**Table 1:** Single-board computer specifications.

Raspberry Pi 3 Model B	
CPU	1.2 GHz 64-bit quad-core ARMv8
Memory	1 GB SDRAM
USB 2.0 ports	4 (via the on-board 5-port USB hub)
On-board storage	32 Gb Micro SDHC
On-board network	10/100 Mbit/s Ethernet, 802.11n wireless, Bluetooth 4.1

## References

[1] Warning S.T. Earthquakes Seismic Waves. https://www.sms-tsunami-warning.com/pages/seismic-waves#.XDf1nGhKiiM.

[2] Wilde-Piórko M., Geissler W.H., Plomerová J., Grad M., Babuška V., Brückl E., Cyziene J., Czuba W., England R., Gaczyński E. (2008). PASSEQ 2006–2008: Passive seismic experiment in Trans-European Suture Zone. Stud. Geophys. Geod..

[3] Song W.Z., Huang R., Xu M., Shirazi B.A., LaHusen R. (2010). Design and Deployment of Sensor Network for Real-Time High-Fidelity Volcano Monitoring. IEEE Trans. Parallel Distrib. Syst..

[4] Huang R., Song W.Z., Xu M., Peterson N., Shirazi B., LaHusen R. (2012). Real-World Sensor Network for Long-Term Volcano Monitoring: Design and Findings. IEEE Trans. Parallel Distrib. Syst..

[5] Upton E. Raspberry Pi 3. https://www.raspberrypi.org/products/raspberry-pi-3-model-b.

[6] Helgerud P., Bragstad H. (1996). Method for Synchronization of Systems for Seismic Surveys, Together With Applications of the Method. U.S. Patent.

[7] Antoniou A. (2016). Digital Signal Processing.

[8] Maxwell S. (2014). Microseismic Imaging of Hydraulic Fracturing: Improved Engineering of Unconventional Shale Reservoirs.

[9] Bensen G.D., Ritzwoller M.H., Barmin M.P., Levshin A.L., Lin F., Moschetti M.P., Shapiro N.M., Yang Y. (2007). Processing seismic ambient noise data to obtain reliable broad-band surface wave dispersion measurements. Geophys. J. Int..

[10] Douglas A. (1997). Bandpass filtering to reduce noise on seismograms: Is there a better way?. Bull. Seismol. Soc. Am..

[11] Li F., Song W. (2017). Automatic arrival identification system for real-time microseismic event location. SEG Technical Program Expanded Abstracts 2017.

[12] Du Z., Foulger G.R., Mao W. (2000). Noise reduction for broad-band, three-component seismograms using data-adaptive polarization filters. Geophys. J. Int..

[13] Nakata N., Chang J.P., Lawrence J.F., Boué P. (2015). Body wave extraction and tomography at Long Beach, California, with ambient-noise interferometry. J. Geophys. Res. Solid Earth.

[14] Allen R.V. (1978). Automatic earthquake recognition and timing from single traces. Bull. Seismol. Soc. Am..

[15] Lin F., Ritzwoller M.H., Snieder R. (2009). Eikonal tomography: surface wave tomography by phase front tracking across a regional broad-band seismic array. Geophys. J. Int..

[16] Valero M., Li F., Wang S., Lin F.C., Song W. (2018). Real-time Cooperative Analytics for Ambient Noise Tomography in Sensor Networks. IEEE Trans. Signal Inf. Process. Netw..

[17] Cal’i M., Ambu R. (2018). Advanced 3D Photogrammetric Surface Reconstruction of Extensive Objects by UAV Camera Image Acquisition. Sensors.

[18] Song W., Shi L., Kamath G., Xie Y., Peng Z. Real-time In-situ Seismic Imaging: Overview and Case Study. Proceedings of the SEG Annual Meeting 2015.

[19] Kamath G., Shi L., Song W.Z., Lees J. (2016). Distributed Travel-time Seismic Tomography in Large-Scale Sensor Networks. J. Parallel Distrib. Comput..

[20] Zhao L., Song W.Z., Shi L., Ye X. (2015). Decentralized Seismic Tomography Computing In Cyber-Physical Sensor Systems. Cyber-Phys. Syst..

[21] Kamath G., Shi L., Chow E., Song W.Z. (2015). Distributed Tomography with Adaptive Mesh Refinement in Sensor Networks. Int. J. Sens. Netw..

[22] Ramanan P., Kamath G., Song W.Z. (2015). INDIGO: An In-Situ Distributed Gossip Framework for Sensor Networks. Int. J. Distrib. Sens. Netw..

[23] Valero M., Kamath G., Clemente J., Lin F.C., Xie Y., Song W. Real-time Ambient Noise Subsurface Imaging in Distributed Sensor Networks. Proceedings of the 3rd IEEE International Conference on Smart Computing (SMARTCOMP 2017).

[24] Shi L., Song W.Z., Dong F., Kamath G. Sensor Network for Real-time In-situ Seismic Tomography. Proceedings of the International Conference on Internet of Things and Big Data (IoTBD 2016).

[25] Kamath G., Shi L., Song W.Z. Component-Average based Distributed Seismic Tomography in Sensor Networks. Proceedings of the 9th IEEE International Conference on Distributed Computing in Sensor Systems (IEEE DCOSS).

[26] Boyd S., Parikh N., Chu E., Peleato B., Eckstein J. (2011). Distributed optimization and statistical learning via the alternating direction method of multipliers. Found. Trends Mach. Learn..

[27] Wu T., Yuan K., Ling Q., Yin W., Sayed A.H. Decentralized consensus optimization with asynchrony and delay. Proceedings of the IEEE Asilomar Conference on Signals, Systems, and Computers.

[28] Aysal T.C., Yildiz M.E., Sarwate A.D., Scaglione A. (2009). Broadcast Gossip Algorithms for Consensus. IEEE Trans. Signal Process..

[29] Matei I., Baras J.S. (2011). Performance Evaluation of the Consensus-Based Distributed Subgradient Method Under Random Communication Topologies. Sel. Top. Signal Process. IEEE J..

[30] Nedic A., Ozdaglar A. (2009). Distributed Subgradient Methods for Multi-Agent Optimization. Autom. Control IEEE Trans..

[31] Yuan K., Ling Q., Yin W. (2013). On the convergence of decentralized gradient descent. arXiv.

[32] Chen A.I.A. (2012). Fast Distributed First-Order Methods. Ph.D. Thesis.

[33] Xiao L., Boyd S., Lall S. A Scheme for Robust Distributed Sensor Fusion Based on Average Consensus. Proceedings of the 4th International Symposium on Information Processing in Sensor Networks.

[34] Tsitsiklis J.N., Bertsekas D.P., Athans M. (1986). Distributed asynchronous deterministic and stochastic gradient optimization algorithms. Autom. Control IEEE Trans..

[35] Terelius H., Topcu U., Murray R. Decentralized multi-agent optimization via dual decomposition. Proceedings of the 18th IFAC World Congress.

[36] Rabbat M., Nowak R. Distributed optimization in sensor networks. Proceedings of the Third International Symposium on Information Processing in Sensor Networks, 2004 (IPSN 2004).

[37] Dusan Jakovetic J.M. (2014). Fast Distributed Gradient Methods. arXiv.

[38] Iutzeler F., Bianchi P., Ciblat P., Hachem W. (2013). Asynchronous Distributed Optimization using a Randomized Alternating Direction Method of Multipliers. arXiv.

[39] Nedic A. (2011). Asynchronous Broadcast-Based Convex Optimization Over a Network. IEEE Trans. Autom. Control.

[40] Zhao L., Song W.Z., Ye X., Gu Y. (2018). Asynchronous Broadcast-based Decentralized Learning in Sensor Networks. Int. J. Parallel Emerg. Distrib. Syst..

[41] Wong J., Han L., Bancroft J., Stewart R. Automatic Time-Picking of First Arrivals on Noisy Microseismic Data. https://www.crewes.org/ForOurSponsors/ConferenceAbstracts/2009/CSEG/Wong_CSEG_2009.pdf.

[42] Baer M., Kradolfer U. (1987). An automatic phase picker for local and teleseismic events. Bull. Seismol. Soc. Am..

[43] Takanami T., Kitagawa G. (1991). Estimation of the arrival times of seismic waves by multivariate time series model. Ann. Inst. Stat. Math..

[44] Anant K.S., Dowla F.U. (1997). Wavelet transform methods for phase identification in three-component seismograms. Bull. Seismol. Soc. Am..

[45] Molyneux J.B., Schmitt D.R. (1999). First-break timing: Arrival onset times by direct correlation. Geophysics.

[46] Li F., Rich J., Marfurt K.J., Zhou H. (2014). Automatic event detection on noisy microseismograms. SEG Technical Program Expanded Abstracts 2014.

[47] Akram J., Eaton D.W. (2016). A review and appraisal of arrival-time picking methods for downhole microseismic dataArrival-time picking methods. Geophysics.

[48] Li S., Cao Y., Leamon C., Xie Y., Shi L., Song W. Online seismic event picking via sequential change-point detection. Proceedings of the 2016 54th Annual Allerton Conference on Communication, Control, and Computing (Allerton).

[49] Baillard C., Crawford W.C., Ballu V., Hibert C., Mangeney A. (2014). An automatic kurtosis-based P-and S-phase picker designed for local seismic networks. Bull. Seismol. Soc. Am..

[50] Lees J.M., Crosson R.S. (1991). Bayesian Art versus Conjugate Gradientf Methods in Tomographic Seismic Imaging: An Application at Mount St. Helens, Washington. Lecture Notes-Monogr. Ser..

[51] Butrylo B., Tudruj M., Masko L. Distributed Formulation of Artificial Reconstruction Technique with Reordering of Critical Data Sets. Proceedings of the Fifth International Symposium on Parallel and Distributed Computing, 2006. ISPDC’06.

[52] Zhang H., Thurber C. (2006). Development and applications of double-difference seismic tomography. Pure Appl. Geophys..

[53] Shi L., Song W.Z., Xu M., Xiao Q., Lees J.M., Xing G. Imaging Volcano Seismic Tomography in Sensor Networks. Proceedings of the 10th Annual IEEE Communications Society Conference on Sensor and Ad Hoc Communications and Networks (IEEE SECON).

[54] Gordon D., Gordon R. (2005). Component-averaged row projections: A robust, block-parallel scheme for sparse linear systems. SIAM J. Sci. Comput..

[55] Censor Y., Gordon D., Gordon R. (2001). Component averaging: An efficient iterative parallel algorithm for large and sparse unstructured problems. Parallel Comput..

[56] Zhao L., Song W.Z., Ye X. Fast Decentralized Gradient Descent Method and Applications to In-situ Seismic Tomography. Proceedings of the IEEE International Conference on Big Data (IEEE BigData 2015).

[57] Artman B. (2006). Imaging passive seismic data. Geophysics.

[58] Wong M., Biondi B.L., Ronen S. (2015). Imaging with primaries and free-surface multiples by joint least-squares reverse time migration. Geophysics.

[59] Sun J., Zhu T., Fomel S., Song W.Z. (2015). Investigating the possibility of locating microseismic sources using distributed sensor networks. SEG Technical Program Expanded Abstracts 2015.

[60] Nakata N., Beroza G.C. (2016). Reverse time migration for microseismic sources using the geometric mean as an imaging condition. Geophysics.

[61] Wu S., Wang Y., Zheng Y., Chang X. (2017). Microseismic source locations with deconvolution migration. Geophys. J. Int..

[62] Kao H., Shan S.J. (2004). The Source-Scanning Algorithm: mapping the distribution of seismic sources in time and space. Geophys. J. Int..

[63] Gajewski D., Tessmer E. (2005). Reverse modelling for seismic event characterization. Geophys. J. Int..

[64] Witten B., Artman B. (2011). Signal-to-noise estimates of time-reverse images. Geophysics.

[65] Kremers S., Fichtner A., Brietzke G.B., Igel H., Larmat C., Huang L., Käser M. (2011). Exploring the potentials and limitations of the time-reversal imaging of finite seismic sources. Solid Earth.

[66] Yavuz M.E., Teixeira F.L. (2008). Space–frequency ultrawideband time-reversal imaging. IEEE Trans. Geosci. Remote Sens..

[67] Yang H., Li T., Li N., He Z., Liu Q.H. (2016). Time-gating-based time reversal imaging for impulse borehole radar in layered media. IEEE Trans. Geosci. Remote Sens..

[68] Zhang Y., Xu S., Bleistein N., Zhang G. (2007). True-amplitude, angle-domain, common-image gathers from one-way wave-equation migrations. Geophysics.

[69] Claerbout J.F. (1985). Imaging the Earth’s Interior.

[70] Araya-Polo M., Cabezas J., Hanzich M., Pericas M., Rubio F., Gelado I., Shafiq M., Morancho E., Navarro N., Ayguade E. (2011). Assessing accelerator-based HPC reverse time migration. IEEE Trans. Parallel Distrib. Syst..

[71] Liu H., Long Z., Tian B., Han F., Fang G., Liu Q.H. (2017). Two-Dimensional Reverse-Time Migration Applied to GPR With a 3-D-to-2-D Data Conversion. IEEE J. Sel. Top. Appl. Earth Obs. Remote Sens..

[72] Schuster G.T., Yu J., Sheng J., Rickett J. (2004). Interferometric/daylight seismic imaging. Geophys.J. Int..

[73] Witten B., Shragge J. (2015). Extended wave-equation imaging conditions for passive seismic data. Geophysics.

[74] Rentsch S., Buske S., Lth S., Shapiro S.A. Location of seismicity using Gaussian beam type migration. Proceedings of the 2004 SEG Annual Meeting.

[75] Wang S., Li F., Song W. Microseismic Source Location with Distributed Reverse Time Migration. http://sensorweb.engr.uga.edu/wp-content/uploads/2018/06/Dropbox_wang2018microseismic.pdf.

[76] Lin F., Moschetti M.P., Ritzwoller M.H. (2008). Surface wave tomography of the western United States from ambient seismic noise : Rayleigh and Love wave phase velocity maps. Geophys. J. Int..

[77] Moschetti M.P., Ritzwoller M.H., Lin F., Yang Y. (2010). Crustal shear wave velocity structure of the western United States inferred from ambient seismic noise and earthquake data. J. Geophys. Res..

[78] Lin F.C., Ritzwoller M.H., Yang Y., Moschetti M.P., Fouch M.J. (2011). Complex and variable crustal and uppermost mantle seismic anisotropy in the western United States. Nat. Geosci..

[79] Roux P., Sabra K.G., Gerstoft P., Kuperman W.A., Fehler M.C. (2005). P-waves from cross-correlation of seismic noise. Geophys. Res. Lett..

[80] Snieder R. (2004). Extracting the Green’s function from the correlation of coda waves: A derivation based on stationary phase. Phys. Rev. E.

[81] Shapiro N.M., Campillo M., Stehly L., Ritzwoller M.H. (2005). High resolution surface wave tomography from ambient seismic noise. Science.

[82] Gouedard P., Roux P., Campillo M. (2008). Small Scale seismic inversion using surface waves extracted from noise cross-correlation. J. Acoust. Soc. Am..

[83] Longuet-Higgins M.S. (1950). A theory of the origin of microseisms. Phil. Trans. R. Soc. Lond. A.

[84] Picozzi M., Parolai S., Bindi D., Strollo (2009). Characterization of shallow geology by high-frequency seismic noise tomography. Geophys. J. Int..

[85] Yang Y., Ritzwoller M.H., Jones C.H. (2011). Crustal structure determined from ambient noise tomography near the magmatic centers of the Coso region, southeastern California. Geochem. Geophys. Geosyst..

[86] Lin F., Li D., Clayton R.W., Hollis D. (2013). High-resolution 3D shallow crustal structure in Long Beach, California: Application of ambient noise tomography on a dense seismic array. Geophysics.

[87] Lobkis O.I., Weaver R.L. (2001). On the emergence of the Green’s function in the correlations of a diffuse field. J. Acoust. Soc. Am..

[88] Lin F., Ritzwoller M.H. (2011). Helmholtz surface wave tomography for isotropic and azimuthally anisotropic structure. Geophys. J. Int..

[89] Valero M., Li F., Li X., Song W. Imaging Subsurface Civil Infrastructure with Smart Seismic Network. Proceedings of the 37th IEEE International Performance Computing and Communications Conference (IPCCC) 2018.

